# What Is the Role of Nutraceutical Products in Cancer Patients? A Systematic Review of Randomized Clinical Trials

**DOI:** 10.3390/nu15143249

**Published:** 2023-07-22

**Authors:** Raffaella Di Napoli, Nunzia Balzano, Annamaria Mascolo, Carla Cimmino, Antonio Vitiello, Andrea Zovi, Annalisa Capuano, Mariarosaria Boccellino

**Affiliations:** 1 Campania Regional Centre for Pharmacovigilance and Pharmacoepidemiology, 80138 Naples, Italy; raffaella.dinapoli@unicampania.it (R.D.N.); nunziabalzano95@gmail.com (N.B.); annalisa.capuano@unicampania.it (A.C.); 2 Department of Experimental Medicine, Section of Pharmacology “L. Donatelli”, University of Campania “Luigi Vanvitelli”, 80138 Naples, Italy; 3 Ordine Biologi Campania Molise, 80133 Naples, Italy; carlacimmino81@gmail.com; 4 Ministry of Health, Viale Giorgio Ribotta 5, 00144 Rome, Italy; avitiello@hotmail.it (A.V.); zovi.andrea@gmail.com (A.Z.); 5 Department of Precision Medicine, University of Campania “Luigi Vanvitelli”, 80138 Naples, Italy; mariarosaria.boccellino@unicampania.it

**Keywords:** nutraceutical products, cancer patients, RCT

## Abstract

Chemotherapy represents the main pharmacological cancer treatment. Recently, positive effects emerged with the combination of anticancer therapy and nutraceutical products. The aim of this systematic review is to collect and synthesize the available scientific evidence regarding the potential effects of nutraceuticals on cancer cells. A systematic literature search of randomized clinical trials of nutraceutical products in patients with cancer published up to 15 December 2022 was conducted using three data sources: Embase, PubMed, and Web of Science. The effect of high-dose isoflavone supplements on prostate cancer resulted in stabilization or reduction of PSA concentrations in 50% of isoflavone group patients six months after treatment. High doses of vitamin D supplementation plus chemotherapy in patients with advanced or metastatic colorectal cancer showed a median PFS of 13.0 months (95% CI, 10.1–14.7 months) for 49 patients. The effect of vitamin D supplementation on markers of inflammatory level and antioxidant capacity in women with breast cancer showed a significant increase in serum vitamin D concentration (28 ± 2.6 to 39 ± 3.5; *p* = 0.004) after 8 weeks of treatment. In conclusion, nutraceutical supplements represent a potentially growing sector and can be utilized in medical treatment or nutrition to provide integrated medical care.

## 1. Introduction

One of the main reasons for global death is cancer. Cancer has extensive physical and emotional effects on the lives of patients. Treatment costs also pose several challenges to healthcare systems and patients. Cancer screening is a form of secondary prevention that reduces tumor progression and cancer-related mortality. The main goals of cancer treatment are to increase quality of life and to prolong survival. Since most cancers are diagnosed with poor prognosis, an early diagnosis and targeted treatment can increase the chances of survival and healing. Several multifactorial pathophysiological factors, such as genetic mutations, infection or inflammation, stress, poor dietary habits, and radiation exposure, can contribute to cancer progression [1]. Cancer is characterized by the uncontrolled growth of cells in any part of the body, in particular, a malignant tumor is made up of cells with infiltrating capacity and motility, with some cancer cells able to detach from their site of origin and travel through the blood or lymphatic system to distant parts of the body and produce metastases [2]. This process is responsible for >90% of tumor-related deaths, often due to the impairment of vital organ function [3].

To date, chemotherapy represents the main pharmacological treatment. However, anticancer drugs have harmful effects on normal cells, carrying the risk of side effects that can sometimes cause serious complications and negate the benefits in terms of hospitalization and survival. Recently, from the literature, positive effects emerged with the combination of anticancer therapy and nutraceutical products. Nutraceuticals are used daily to supplement nutrients that are lacking in the diet [4].

Bioactive phytochemicals, such as alkaloids, various terpenoids, and polyphenols (including anthocyanins, flavones, flavanols, isoflavones, stilbenes, ellagic acid, and others), are an important source of nutraceutical ingredients. These phytochemicals are mainly produced by plants and serve as non-essential nutrients with either defensive or disease-protective properties. Phytochemicals can have specific pharmacological effects, such as antioxidant, anti-inflammatory, chemo-preventive, hypotensive, and anti-aging effects [5].

Several nutraceuticals, such as vitamins, probiotics, or nutritional support supplements, associated with conventional treatments can contribute to the success of anticancer therapy by neutralizing cancer cells without causing toxicity.

Vitamins such as vitamin C and vitamin D have been studied for their potential to improve cancer outcomes. In a subgroup of patients with early-stage adenocarcinoma and low vitamin D levels, oral vitamin D supplementation significantly increased recurrence-free survival (RFS) and overall survival (OS) [6].

Regarding vitamin C, its supplementation did not reduce the post-trial follow-up period in colorectal cancer patients, suggesting a possible late effect of vitamin C supplementation [7].

In immunosuppressed patients with advanced colorectal cancer, vitamin E also showed a beneficial effect by restoring patients’ antioxidant status and improving NK cell function [8].

Probiotics such as Lactobacillus acidophilus and Bifidobacterium bifidum have been suggested to have anti-cancer properties and can help to reduce the side effects of chemotherapy and radiation therapy. Nutritional support supplements, such as omega-3 fatty acids, curcumin, and resveratrol, have also been studied for their potential to support anticancer therapy [9,10,11]. Different cellular and extracellular biochemical mechanisms can explain their effects on the survival of neoplastic cells, including epigenetic mechanisms that modulate the expression of genes involved in the stages of tumor promotion and progression [12,13,14]. In addition, some natural substances can counteract the inflammatory process that predisposes cells to carcinogenesis and protect cells by producing oxidative stress-inducing anti-proliferative effects in different types of neoplasia [2,15]. On the other hand, emerging opinions have highlighted that high levels of a nutraceuticals may compromise the effects of chemotherapy, making cancer cells less sensitive to treatment [12]. However, no systematic reviews and meta-analysis of randomized clinical trials have been conducted to evaluate the effectiveness of nutraceutical compounds and their safety profile in cancer patients. Therefore, our systematic review aims to provide an overview of published randomized trials on the potential effects of dietary supplements on cancers and to provide robust research evidence for their safety and efficacy.

## 2. Methods of the Systematic Review

### 2.1. Protocol Registration and Reporting Format

We registered the review in the PROSPERO database with the identification number CRD42023398028, hosted by the National Institute for Health Research, University of York, Center for Reviews and Dissemination, available at: https://www.crd.york.ac.uk/prospero/display_record.php?RecordID=398028 (accessed on 1 February 2023).

To carry out a standardized search of data and extraction, as well as reporting and presentation, we used the Preferred Reporting Items for Systematic Reviews and Meta-analysis (PRISMA) guidelines [16]. A systematic literature search of randomized clinical trials (RCTs) of nutraceutical products in patients with cancer published up to 15 December 2022, was conducted using the following three data sources: Embase, PubMed, and Web of Science.

### 2.2. Search Strategy and Selection Criteria

Two investigators (RDN and NB) systematically searched the scientific literature. One of them developed inclusion criteria and analyzed titles and abstracts of emerging studies (RDN) by selecting them based on their study design. At this stage, only RCTs were selected and were screened in the second stage by another investigator (NB). The other investigator also evaluated the selection criteria and independently analyzed retrieved articles. In case of contradictions, the opinion of a third investigator was sought (AC). In the second stage, full-text articles of RCTs were screened to select only those evaluating the efficacy and safety of nutraceutical products. The search results were downloaded from databases and inserted in an Excel file for the removal of duplicates. The search terms were: ((“nutraceutic*” or “nutraceutical*” or “functional foods” or “dietary supplements”) and (“efficacy” or “effectiveness” or “effectiv*” or “safety” or “adverse event” or “reaction” or “tolerability”) and (“random*” or “clinical trial” or “interventional”) and (“tumor” or “tumour” or “cancer” or “malignan*”)).

### 2.3. PICO Question

We included articles that met the PICOS criteria (population, intervention, comparator, outcome, and study): (I) the study population included patients diagnosed with cancer; (II) intervention defined as a group who received a nutraceutical product; (III) the comparator defined as patients receiving an alternative nutraceutical, placebo, or no intervention; (IV) primary efficacy outcome defined as a reduction of tumor or inflammatory biomarkers and/or OS, progression-free survival (PFS), tumor objective response rate, overall response rate (ORR), disease control rate (DCR), RFS, time to progression (TTP). Secondarily, the safety was evaluated for the retrieved articles; (V) study design defined as interventional, randomized, controlled clinical trial.

### 2.4. Eligibility Criteria

Randomized clinical trials that considered the efficacy and safety of nutraceutical products were included. Observational studies, meta-analyses, letters, case reports, editorials, clinical trial reviews, meeting abstracts, posters, protocols, and books were excluded. Finally, studies not considering our outcomes, and articles not in the English language, were excluded.

### 2.5. Study Evaluation

Articles were assessed for the study population, type of nutraceutical products and their dosage, comparison, sample size, and cancer stage.

## 3. Results of the Systematic Review

**Figure 1:**
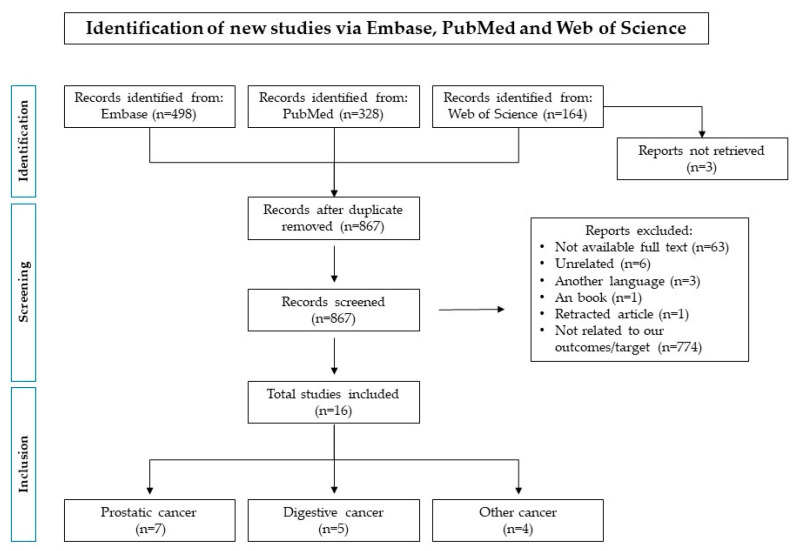
Flow-chart for the identification of eligible randomized clinical trials.

A total of 990 records were initially identified, of which 498 were retrieved from Embase, 328 from PubMed, and 164 from Web of Science. After the duplication removal, a total of 867 records were screened. 774 were excluded because they were not related to our outcomes/target/study design, 63 were not available as full-text, six were unrelated, three were in another language, one was part of a book and one was retracted. As a result, 16 studies were included. A complete representation of the screening process, including studies excluded for each step, is reported in Figure 1.

We organized them into three groups: prostatic cancers, digestive cancers, and other cancers. Characteristics of included RCTs are shown in Table 1, Table 2 and Table 3.

### 3.1. Randomized Clinical Trials Focused on Prostatic Cancer

Six RCTs which referred to prostatic cancer (PCa) were identified.

Gontero P et al. conducted a Phase I-II RCT to evaluate the effect of dietary supplements containing lycopene, selenium, and green tea catechins (GTCs) compared to placebo in patients with multifocal high-grade prostatic intraepithelial neoplasia (mHGPIN) and/or atypical small acinar proliferation (ASAP). Phase I enrolled ten patients to take supplementation or placebo for one month and evaluated the chemical stability, tolerability, and blood concentrations of lycopene. Another 50 patients were included in Phase II for a total of 60 men randomized into two groups to evaluate the disease (PCa and/or HGPIN/ASAP incidence) at re-biopsy in the two groups. Variations of PSA, international prostate symptom score (IPSS), and microRNA (miRNA) expressions were secondary endpoints. They also performed pre- and post-treatment molecular analyses comparing miRNA levels in tissue samples adjacent to pre- and neoplastic lesions. All parameters monitored at randomization were further analyzed at the 6-month follow-up visit. No significant difference was observed in the average age of patients, which for the overall population was 63.3 years (SD 7). Six months after treatment at re-biopsy, 13 men (24.5%) were diagnosed with PCa (supplementation *n* = 10, placebo *n * =  3 [*p* = 0.053]), and a stronger modulation of miRNAs was observed in the nutraceutical group compared to the placebo. Overexpression of miRNAs present in PCa compared to non-cancerous tissue was found, followed by an underexpression of miRNAs suppressing PCa proliferation. In PCa, men also reported the detection of 35 miRNAs, including androgen-regulated miR-125b-5p and PTEN-targeting miR-92a-3p (both upregulated) compared to disease-free patients. No significant difference in prostate specific antigen (PSA) and IPSS was observed [19].

Another RCT conducted by deVere White RW et al. evaluated the effect of high-dose isoflavone supplements on PCa. The study was conducted over 12 months and was divided into two periods. From zero to six months the study was double blind, and from six to twelve months the study was open label. In the double-blind study, the stabilization or reduction of PSA concentrations was found in 50% of patients in the GCP (mixture of isoflavone) group (14/28) and in 32% of the placebo group (8/25). Among the safety outcomes of GCP treatment, loose stools were the most common adverse reaction reported; however, the high intake of aglycone isoflavones in this RCT was well tolerated. In the open-label study, no difference between groups (*p*-value: 0.915) was observed for PSA concentrations, although an increase was found in the placebo group. Metastases were not detected in any patient [18].

Chan JM et al. evaluated the effects of lycopene and fish oil supplements versus placebo among men receiving active surveillance for low-burden PCa. This study included three treatment arms: lycopene + placebo for fish oil (lycopene arm); fish oil + placebo for lycopene (fish oil arm); or placebo for lycopene + placebo for fish oil (placebo arm). In addition, everyone also received a standard daily multivitamin. The primary outcomes were changes in gene expression of the insulin-like growth factor-1 (IGF-1) and cyclooxygenase 2 (COX-2) among biopsies (up to 3 months). A total of 69 men were enrolled (22 lycopene group, 21 fish group, and 26 placebo group). There was no difference in the IGF-1 or IGF-1R expressions between the placebo and the lycopene arms (*p* = 0.93 and *p* = 0.53) after 3 months. There was also no difference in COX-2 expression between the placebo group and the fish group (*p* = 0.99). In terms of adverse events, two patients reported indigestion and one patient had migraine in the lycopene group. These events were classified as “possibly related” [17].

Grainger EM et al. evaluated the effect of tomato and soy products on the increased risk of PCa or enhancement in therapeutic efficacy. Patients were divided into two groups. Group A received tomato and Group B received soy for four weeks. All patients then received a combination of soy and tomato products for the next four weeks. During the study period, there were no significant changes in IGF-1 and testosterone levels in either group. The serum vascular endothelial growth factor (VEGF) concentrations analyzed at weeks 0, 4, and 8 were significantly reduced between weeks 0 and 8 (*p* < 0.04) in all patients. Specifically, VEGF levels were 87 ± 126 ng/mL at week 0; 55 ± 43 mg/mL at week 4; and 51 ± 35 ng/mL at week 8. Moreover, a lower PSA at the end of the study was found in Group A 5/20 (25%) and Group B 9/21 (43%). Considering adverse events, constipation and a flare-up of gout were reported in 7% and 2% of patients taking the soy protein supplement, respectively [20].

The RCT of Kumar NB et al. evaluated the safety and effectiveness of purified isoflavones compared to placebo in the modulation of steroid hormones in men with PCa. This trial showed a significant increase in plasma levels of isoflavone in the treatment group (daidzein *p* < 0.0001; glycitein *p* = 0.01; genistein *p* < 0.0001) from baseline to 12 weeks compared to placebo. Moreover, a not statistically significant reduction of serum testosterone levels was observed with isoflavones compared to the control group (*p* = 0.3). Furthermore, no increase in serum levels of the sex hormone binding globulin (SHBG) in the isoflavones group was detected. Moreover, a decrease in the total estradiol was observed in both groups. The gastrointestinal and metabolic events were similar in the two groups and were all classified as Grades I to II [22].

In another study, Kumar NB et al. analyzed the effectiveness of a soy isoflavone supplement on changes of hormonal levels in the early stages of PCa. Seventy-six patients with PCa aged between 45 and 85 years were enrolled. The results showed a decrease or no change in serum free testosterone in 61% of patients treated with isoflavone supplement compared to 33% of patients treated with placebo. However, these differences were not statistically significant. Moreover, a decrease or no change in PSA level was observed in 69% of patients treated with isoflavone supplement and in 55% of those treated with placebo. No differences in the increase of total estradiol and SHBG were found (*p* = 0.91 and *p* = 0.97, respectively [21].

Finally, Schröder FH et al. conducted a crossover study evaluating the effect on the increased rate of PSA of a dietary supplement (soy, isoflavones, lycopene, silymarin and antioxidants as main ingredients) compared to placebo. Patients with increased PSA after radical prostatectomy or curative radiation therapy were included. Results showed an improvement in the slope of the 2log transformed PSA concentrations with the supplement treatment compared to placebo, both in the intention-to-treat (ITT) and the per-protocol (PP) analyses. The PP population also showed a statistically significant estimate (*p* = 0.041) [23].

Thus, most studies did not show a potential benefit of the use of nutraceuticals in PCa patients. The current evidence cannot be used to draw valid conclusions about the efficacy of these nutraceutical supplements. Therefore, a better understanding of these anti-cancer mechanisms would be beneficial and further well-powered studies should be conducted.

### 3.2. Randomized Clinical Trials Focused on Digestive Cancer

Five RCTs focused on digestive cancers. One conducted by Urashima M et al. aimed to assess the efficacy of vitamin D supplementation in terms of RFS and OS among patients with digestive tract cancers after surgical resection, including esophagus, stomach, small intestine, colon, and rectum cancers. It was conducted at a single university hospital in Japan and enrolled 417 patients. The study showed that vitamin D supplementation compared with placebo did not result in a significant improvement in RFS [77% vs. 69%; (hazard ratio, HR, for relapse or death, 0.76; 95% CI, 0.50–1.14; *p* = 0.18)] and OS [82% vs. 81%; (HR for death, 0.95; 95% CI, 0.57–1.57; *p* = 0.83)] after 5 years of treatment. However, in a subgroup of patients with middle serum vitamin D levels (20–40 ng/mL) at baseline, RFS was significantly higher in the group with vitamin D supplementation compared to the placebo (85% vs. 71%; HR for relapse or death, 0.46; 95% CI, 0.24–0.86; *p* = 0.02). Therefore, vitamin D was effective in the middle-baseline-level subgroup. Conversely, there was no significant difference for RFS (HR, 1.15; 95% CI, 0.65–2.05) in a subgroup of patients with low serum vitamin D levels (<20 ng/mL) at baseline. Regarding OS, no statistically significant difference between the vitamin D and placebo groups was found in both the middle-baseline-level subgroup (HR for death, 0.60; 95% CI, 0.28–1.30) and the low-baseline-level subgroup (HR for death, 1.36; 95% CI, 0.66–2.81). This study also highlighted the safety of vitamin D compared with placebo in terms of frequent adverse events. During the follow-up period, no patients developed hypercalcemia. Three patients (1.3%) in the vitamin D group and five patients (3.4%) in the placebo group developed fractures;, while urinary stones occurred in two patients (0.9%) treated with vitamin D supplementation [27].

Another RCT conducted by Ng K et al. evaluated the effect on PFS (disease progression or death) of a high dose of vitamin D supplement added to standard chemotherapy, compared to a standard dose of vitamin D supplementation plus chemotherapy, among patients with advanced or metastatic colorectal cancer. The median PFS was 13.0 months (95% CI, 10.1–14.7 months) for 49 patients in the high-dose vitamin D group compared with 11.0 months (95% CI, 9.5–14.0 months) for 62 patients in the standard-dose vitamin D group. The HR for PFS was 0.64 (95% CI, 0–0.90; *p* =  0.02). The study also showed the results between high-dose and standard-dose vitamin D for tumor objective response rate (58% vs. 63%, respectively; difference, −5% [95% CI, −20% to 100%], *p* = 0.27) or OS (median, 24.3 months vs. 24.3 months; log-rank *p* = 0.43), without any statistically significant difference. In terms of safety, neutropenia (*n* = 24 [35%] vs. *n* = 21 [31%], respectively) and hypertension (*n* = 9 [13%] vs. *n* = 11 [16%], respectively) were higher in patients treated with high-dose vitamin D compared to standard-dose vitamin D supplements. Fewer episodes of diarrhea were reported in the high-dose vitamin D group compared to eight events of diarrhea in the standard-dose vitamin D group. No patients enrolled in this study reported hypercalcemia [25].

Farsad-Naeimi A et al. conducted an RCT to investigate whether fisetin supplementation received for seven consecutive weeks could improve inflammatory status in colorectal cancer patients undergoing chemotherapy. Plasma levels of interleukin (IL)-8, matrix metalloproteinase (MMP)-7 and hs-CRP decreased significantly in the fisetin group (*p* < 0.04, *p* < 0.02 and *p* < 0.01, respectively). However, no significant changes in plasma levels of IL-10 and MMP-9 were found [24].

Another RCT conducted by van Zweeden AA et al. aimed to evaluate folic acid and vitamin B12 supplementation on the efficacy of cisplatin and gemcitabine in patients with advanced esophagogastric cancer. In particular, this study was focused on the results of response rate (RR), OS, or TTP of cisplatin and gemcitabine esophagogastric cancer patients. The RR did not significantly differ between patients supplemented with folic acid and vitamin B12 and unsupplemented patients (42.1% and 32.4%, respectively; *p* = 0.4). The median OS was similar in both groups of patients (10.0 months for supplemented patients and 7.7 months for unsupplemented patients, respectively; *p* = 0.9). The median TTP was not significantly different after vitamin supplementation: 5.9 months (1.4–33.5) with vitamin supplementation and 5.4 months (1.4–30.9) without vitamin supplementation (*p*  =  0.9). The incidence of grade 3–5 adverse events did not appear reduced by vitamin supplementation. In supplemented patients, the most common adverse event was grade 3 leukopenia (*n* = 9 [22%]), whereas fatigue was the most common adverse event (*n* = 10 [24%]) in non-supplemented patients. Moreover, three patients in the supplemented group and one patient in the unsupplemented group developed grade 4 thrombopenia [28].

Finally, Tsai HL et al. conducted an RCT to investigate the efficacy of low-molecular-weight fucoidan, a widely used food supplement, in addition to chemotherapy in metastatic colorectal cancer patients. In the fucoidan group, the DCR was significantly higher than in control patients treated with cellulose powder (92.8% and 69.2%, respectively; *p* = 0.026). Low-molecular-weight fucoidan supplementation led to a small increase in the ORR, but this was not statistically significant (60.7% and 46.2%, respectively; *p* = 0.284). The OS (18.04 ± 0.91 vs. 12.96 ± 0.83 months; *p* = 0.092) and PFS (15.93 ± 1.20 vs. 10.80 ± 1.06 months; *p* = 0.075) did not differ significantly between the two groups. During the trial, there were no severe adverse events observed in either group and no death was observed with the fucoidan treatment. The incidence of oral mucositis (65.4% vs. 50%; *p* = 0.253), pruritus (53.9% vs. 35.7%; *p* = 0.180), vomiting (53.9% vs. 35.7%; *p* = 0.180), taste problems (80.8% vs. 64.3%; *p* = 0.177), and bloody stool (30.8% vs. 14.3%; *p* = 0.145) were higher in patients treated with cellulose powder compared to patients in the treatment with fucoidan supplement. Quality of life evaluated through limitation of daily activities, limitation of walking, anxiety, fatigue, weakness, and issues of personal hygiene was similar between groups, without any statistical difference [26].

These studies did not show a potential role for vitamin D or other nutraceuticals in the treatment of gastro-intestinal cancers in terms of either mortality or inflammation. Collectively, these RCTs did not find potent effects on immune function and inflammation for vitamin supplementation; therefore, not providing any evidence for their use as promising supportive anti-cancer agents.

### 3.3. Randomized Clinical Trials Focused on Other Cancer

Four RCTs related to other cancers were included in our review. Two of them considered the use of nutraceutical products in breast cancer, another one in head and neck cancer, and the last one in skin melanoma. Mohseni H et al. evaluated the effect of vitamin D supplementation compared to placebo on inflammatory level markers and antioxidant capacity in women with breast cancer. Significant increases in serum concentrations of vitamin D (28 ± 2.6 to 39 ± 3.5; *p* = 0.004) and TAC (48.9 ± 13.3 to 63.5 ± 13.3; *p* = 0.017) were reported in the supplementation group after 8 weeks of treatment. Variations of TGF-β1 and TNF-α were not statistically significant between groups. The total antioxidant capacity levels of participants with the TT/Tt, Ff genotypes was improved in patients with supplementation [31].

Another RCT conducted by Shahvegharasl Z et al. evaluated the effects of vitamin D supplementation compared to placebo on serum levels of angiogenic parameters in breast cancer patients treated with tamoxifen. Serum levels of angiopoietin (Ang)-2, hypoxia-inducible factor (Hif)-1, high-sensitivity C-reactive protein (hs-CRP), and Ang-2/VEGF-A were increased during the treatment period. After 8 weeks of treatment, premenopausal women had shown a significant decrease in serum levels of Ang-2 and VEGF-A (*p* < 0.05). In patients with infiltration of tumor into lymphatic and vascular vessels, a significant increase of Hif-1 emerged (*p* < 0.05). No adverse effects related to vitamin D supplementation were reported in this trial [32].

Datta M et al. analyzed the effect of Juice PLUS+ (JP; a commercial product with multiple FV concentrates) on p27 (a cyclin-dependent kinase inhibitor) and Ki-67 (cell proliferation associated nuclear protein), which are biomarkers related to the risk of second primary tumors (SPTs). In the JP group, 12 weeks later, significantly higher serum levels of α-carotene (*p* = 0.009), β-carotene (*p* < 0.0001), and lutein (*p* = 0.003), but not of p27 (*p* = 0.23) or Ki-67 (*p* = 0.95) were observed. The SPT prevention was not significantly connected with continuous consumption after the initial 12 weeks of JP. Seven adverse events, including anorexia, nausea, vomiting, diarrhea, fatigue, fever, and heartburn were reported (JP = 3; placebo = 4). The most common toxicity was heartburn (13%). All other events (frequency < 7%) were the same between study groups [29].

Finally, Demidov LV et al. reported the adjuvant use of Fermented Wheat Germ Extract (Avemar™) in the treatment of high-risk skin melanoma patients. During the follow-up period, a mean PFS of 55.8 months was found in the fermented wheat germ extract (FWGE) group compared to 29.9 months in the control group (*p* = 0.0137). The mean OS was 66.2 months for FWGE group and 44.7 months for the control group (*p* = 0.0298). Fewer adverse events emerged in patients who received the combined therapy compared to the control group. All toxicity in the FWGE group was transient and mild [30].

Most studies on other types of cancers also failed to demonstrate the potential efficacy of nutraceutical products. Taken together, the little evidence does not suggest a use for breast cancer, head and neck cancer, and skin melanoma. Nevertheless, the limited literature on this topic acknowledges that a higher number of patients enrolled, and more types of cancer investigated, are needed to strengthen evidence on the potential effects of nutraceutical products on cancer patients and improve the quality of results. For this reason, based on the current knowledge, it is premature to make conclusions on the administration of nutraceutical products in addition to chemotherapy.

## 4. Discussion

Our systematic review of RCTs explored the efficacy and safety of nutraceuticals as supportive therapy for many cancers. The aim of this review was to provide further evidence regarding the use of nutraceuticals in addition to primary chemotherapy or radiotherapy in cancer patients, and to support the current interest among clinicians and consumers in this area. Nutraceutical classes, such as polyphenolic compounds, carotenoids, polyunsaturated fatty acids and vitamins, may help in cancer therapy through various mechanisms of action (Table 4).

In particular, the literature suggests that the anticancer effects of nutraceuticals are probably related to their capacity for changing signal pathways leading to cell growth or cell death by inhibiting cellular proliferation, angiogenesis, and invasion, and by inducing apoptosis [2]. For this reason, it is important to highlight the potential synergies of nutraceutical products in combination with immunotherapy. It is known that immunotherapies improve the immune system’s ability to recognize and eliminate cancer. Cancer cells could be more susceptible to chemotherapy when nutraceuticals are combined with immunotherapy. This may be linked to the ability of natural products to recognize and target mutant genes, modify signal pathways, reduce cancer cell growth, tumor progression and metastasis. This synergism can increase the immunotherapy’s effectiveness in terms of the reduction of drug dosage and resistance and decrease the side effects of chemotherapy [37]. New findings related to isoflavones and lycopene highlighted a lower PCa incidence in supplemented patients. This result is generally consistent with the results of our analyzed RCTs focused on PCa. Development of PCa can be induced by the interaction between estrogen and its receptor, through epigenetic modifications, direct genotoxicity, hyperprolactinemia, inflammation, and immunologic changes [38]. Lycopene is a carotenoid presents in tomato products and other red fruits with antioxidant effects and anticancer properties [39]. Regarding variation of PSA, we observed in PCa patients treated with isoflavones a reduction in PSA levels. In particular, a statistically significant reduction of PSA levels after 1 year of isoflavonoid supplementation was observed in people with PCa. It is likely that this can be attributed to two mechanisms of action. The first involves the direct inhibition of 5-alpha-reductase, such as the mechanism of action of finasteride/ dutasteride. The second one is related to the activation of the uridine diphosphate glucuronyl transferase by phytoestrogens, which can transform testosterone into two ineffective metabolites [40]. On the other hand, regarding lycopene, our results showed a significant reduction of PSA levels after a diet rich in tomatoes, probably because lycopene has a synergistic effect with other compounds present in this food, especially glycoalkaloids (tomatine), phenolic compounds (quercetin, kaempferol, naringenin, chlorogenic acid), salicylates, and carotenoids. According to the literature, lycopene can act on cancer through three possible mechanisms: preventing oxidative DNA damage thanks to its antioxidant effect, overexpression of tumor suppressor proteins, and the inhibition of growth and differentiation factors in PCa cells [41]. However, evidence from RCTs is required in order to make recommendations regarding the use of lycopene and isoflavonoid supplementation in PCa patients.

Recent evidence highlights the role of vitamin D as an anticancer agent, particularly in the biological mechanisms that arrest the cell cycle, induce apoptosis, inhibit inflammation, and repress pathologic angiogenesis [42]. In one of our RCTs focused on breast cancer, a reduction in Ang-2 and VEGF-A levels was observed in premenopausal women after cholecalciferol supplementation. This reduction could be due to a decrease in aromatase expression induced by vitamin D. Aromatase catalyzes estrogen synthesis selectively from androgen in breast cancer cells. The binding between estrogen and its receptor induced the proliferation and growth of cancer cells [43]. To date, there is limited evidence about the role of vitamin D in breast cancer. Other tumors investigated with the vitamin D supplementation were those related to the digestive tract (esophagus, stomach, small intestine, colon, and rectum). We found that vitamin D supplementation did not significantly change the OS, while a meta-analysis of 64 observational studies found a statistically significant difference [44]. Thus, more clinical trials to investigate the potentially beneficial effect of vitamin D in cancer patients are needed. Regarding head and neck cancer, from our results there emerged no evidence about the efficacy of juice plus supplementation, probably due to the lack of RCTs in this area. In the treatment of skin cancer, FWGE supplementation in addiction to chemotherapy (DTIC) was found to be superior to DTIC alone in terms of PFS and OS. Many studies have explained this result as being due to the interaction between DTIC and FWGE when used synchronously. Preclinical experiments have investigated the FWGE’s mechanism of action, which involves the inhibition of the DNA repair enzyme, poly (ADP-ribose) polymerase, which is overexpressed in cancer cells. On the other hand, DTIC inhibits DNA synthesis and cell growth independently from the cell cycle. Therefore, the parallel use of DTIC and FWGE resulted in better synergistic efficacy [45].

To date, there is a lack of research on nutraceuticals and their influence on the processes involved in the progression of common tumors. It is necessary for cancer patients to carefully assess the potential risks and benefits with their healthcare professionals and caregivers before starting treatment with such products.

## 5. Limitations

Although this review provided some interesting observations, its limitations must be highlighted. Firstly, the low number of clinical trials retrieved, since only sixteen RCTs met our selection criteria. Moreover, the high heterogeneity of selected trials, in terms of the numbers of participants included, and the variability in the duration of treatment and in the nutraceutical products administered, did not allow a meta-analysis to be conducted.

## 6. Conclusions

In conclusion, nutraceutical supplements represent a potentially growing sector and can be utilized in medical treatment or nutrition to provide integrated medical care. However, this review does not provide high-quality evidence regarding the efficacy and safety of nutraceuticals for cancer patients. There is a considerable heterogeneity in the type of nutraceutical supplements, outcomes, treatment duration, and dosing reported in the RCTs, suggesting a need to develop well-designed and well-powered clinical trials. To date, it must be emphasized that there is no clinical evidence to support the use of nutraceutical supplements in cancer patients. Furthermore, even our current review provides different and contrasting results regarding the association between each type of supplement and each type of cancer. For this reason, the potential beneficial or destructive effects of supplementation on human health should be explored in future research.

## Figures and Tables

**Table 1:** Characteristics of prostatic cancer patients in randomized clinical trials on nutraceutical supplements.

Author (Year)	Number of Patient	Stage/Grade Prostatic Tumor	Nutraceutic Arm (Type; N)	Control Arm (Type; N)	Age Nutraceutic Arm Mean ± SD	Age Control Arm Mean ± SD	Nutraceutic Dosage	Outcome Included	Results
Chan JM, et al., 2010 [17]	97	- Low-burden prostate cancer - Gleason score of six or lower	First arm: lycopen; *n* = 22 Second arm: oil fish; *n* = 21	Placebo; *n* = 26	First arm: 61 ± 7 Second arm: 62 ± 8	59 ± 8	First arm: two 15 mg lycopene soft gel capsules daily Second arm: three 1 g fish oil capsules daily (including 1098 mg EPA and 549 mg DHA	- Gene expression in putative cancer-related pathways (IGF-1, IGF-1R, and COX-2)	- There was no difference in the IGF-1 or IGF-1R levels (*p* = 0.93 and *p* = 0.53, respectively) after 3 months - There was also no difference in the COX-2 expression (*p* = 0.99)
deVere White RW, et al., 2014 [18]	53	-	GCP; *n* = 28	Placebo; *n* = 25	70.5 ± 9.3	68.6 ± 7.3	5 g/day of GCP, which contained 450 mg genistein and 300 mg daidzein and other isoflavones	- PSA	- The stabilization or reduction of PSA concentrations was found in the 50% (14/28) vs. the 32% (8/25) six months after the treatments
Gontero P, et al., 2015 [19]	53	- High grade prostatic intraepithelial neoplasia (HGPIN) and/or atypical small acinar proliferation (ASAP)	Selenium, lycopene, green tea catechins; *n* = 27	Placebo; *n* = 26	64.1 ± 5.7	62.6 ± 8.2	55 μg selenium; 35 mg lycopene; 600 mg green tea catechins	- Assessment of disease (PCa and/or HGPIN/ASAP incidence) at re-biopsy - Variations of PSA, IPSS - Evaluation of miRNA	- Six months after treatment at re-biopsy, 24.5% were diagnosed with PCa, and a stronger modulation of miRNAs was observed - No significant difference in PSA and IPSS was found - Overexpression of miRNAs in PCa, followed by an underexpression of miRNAs suppressing PCa proliferation
Grainger EM, et al., 2008 [20]	41	-	Group A: First period tomato only, Second period Tomato +soy; *n* = 20	Group B: First period Soy only, Second period Tomato +soy; *n* = 21	-	-	Group A First period tomato only (Lycopene mg/day ± SD 43 ± 15) Second period Tomato +soy (Lycopene mg/day ± SD 40 ± 17; Soy protein g/day ± SD) Group B First period Soy only (protein g/day ± SD 39 ±1) Second period Tomato +soy (Lycopene mg/day 36 ± 11 Soy protein g/day ± SD 39 ± 2)	- Biochemical measures associated with prostate cancer progression including PSA, testosterone, VEGF, and IGF-1	- VEGF concentrations were reduced between weeks 0 and 8 (*p* < 0.04) - A lower PSA was found at the end of the study than at enrollment [Group A 5/20 (25%); Group B 9/21 (43%)] - No change in testosterone or IGF-1 for both groups
Kumar NB, et al., 2004 [21]	76	- Grade 1–2 prostate cancer - Early stage prostate cancer - Gleason score of 6 or below	Isoflavone; *n* = 39	Placebo; *n* = 37	72.5 ± 5.0	70.9 ± 5.3	60 mg/day of genistein	- Levels of free testosterone, - sex-hormone-binding globulin, estradiol, and PSAs	- There was a decrease or no change in serum free testosterone (61% vs. 33%) - No statistically significant increase in the total estradiol and SHBG (*p* = 0.91 and *p* = 0.97, respectively) was observed - Decrease or no change of PSA in 69% vs. 55%
Kumar NB, et al., 2007 [22]	53	- Grade 1–2 prostate cancer - Early stage prostate cancer - Gleason Score of 2–6	Isoflavones; *n* = 25	Placebo; *n* = 28	71.75 ± 6.39	71.92 ± 5.59	80 mg daily	- Levels of isoflavones (daidzein, glycitein, and genistein), steroid hormones such as free testosterone, and SHBG and total estradiol	- There was a significant increase in plasma levels of isoflavone in the treatment group (daidzein *p* < 0.0001; glycitein *p* = 0.01; genistein *p* < 0.0001) from baseline to 12 weeks - Reduction of testosterone levels (*p* = 0.3) - No increase in the SHBG levels for both two groups (*p* = 0.97) - Reduction of total estradiol in both groups (*p* = 0.37)
Schröder FH, et al., 2005 [23]	42	-	Soy, isoflavones, lycopene, silymarin and antioxidant; *n* = 20	Placebo; *n* = 22	-	-	Two tablets of the dietary supplement per day	- Changes in the rate of increase of PSA	- There was an improvement in the rate of increase of 2log transformed PSA concentrations

Abbreviation is as follows: ASAP, atypical small acinar proliferation; COX-2, cyclooxygenase 2; DHA, docosahexaenoic acid; EPA, eicosapentaenoic acid; GCP, genistein combined polysaccharide; HGPIN, highgrade prostatic intraepithelial neoplasia; IGF-1, insulin-like growth factor-1; IPSS, international prostate symptom score; miRNA: microRNA; PCa, prostatic cancer; PSA, prostate specific antigen; SHBG, sex hormone binding globulin; VEGF, vascular endothelial growth factor.

**Table 2:** Characteristics of digestive cancer patients in randomized clinical trials on nutraceutical supplements.

Author (Year)	Number of Patient	Stage Tumor	Nutraceutic Arm (Type; N)	Control Arm (Type; N)	Male/Female Nutraceutic (N)	Male/Female Control (N)	Age Nutraceutic Arm Median (Range)	Age Control Arm Median (range)	Cancer Type	Nutraceutic Dosage	Outcome Included	Results
Farsad-Naeimi A, et al., 2018 [24]	37	Stages II or III	Fisetin; *n* = 18	Placebo; *n* = 19	13/5	10/9	53.87 ± 17.23 *	57.12 ± 14.09 *	Colorectal cancer	100 mg/day	- Levels of IL-8, IL-10, hs-CRP, MMP-7, and MMP-9	- Plasma levels of IL-8, MMP-7 and hs-CRP decreased significantly in the fisetin group (*p* < 0.04, *p* < 0.02 and *p* < 0.01, respectively). - Significant changes in plasma levels of IL-10 and MMP-9 for patients treated with fisetin did not result
Ng K, et al., 2019 [25]	139	-	High-Dose Vitamin D; n *=* 69	Standard-Dose Vitamin D; *n* = 70	41/28	38/32	54 (47–65)	56 (50–64)	Advanced or Metastatic Colorectal Cancer	8000 IU/day (two 4000 IU capsules) for cycle 1 followed by 4000 IU/day for subsequent cycles	- PFS - Tumor ORR - OS	- PFS: [13.0 months (95% CI, 10.1–14.7 months) vs 11.0 months (95% CI, 9.5–14.0 months)] - (HR 0.64 (95% CI, 0–0.90; *p* = 0.02). - Tumor ORR: (58% vs 63%, respectively; difference, −5% [95% CI, −20% to 100%], *p* = 0.27) - OS: (median, 24.3 months vs 24.3 months; log-rank *p* = 0.43)
Tsai HL, et al., 2017 [26]	54	Stage IV	Low-Molecular-Weight Fucoidan; *n* = 28	Placebo; *n* = 26	16/12	15/11	57.46 (30–79)	62.38 (43–83)	Metastatic Colorectal Cancer	4 g twice a day	- DCR - ORR - PFS - OS	- DCR: [92.8% vs. 69.2%, (*p* = 0.026)] - ORR: [60.7% vs. 46.2%, (*p* = 0.284)] - PFS [15.93 ± 1.20 vs. 10.80 ± 1.06 months; (*p* = 0.075)] - OS: [18.04 ± 0.91 vs. 12.96 ± 0.83 months; (*p* = 0.092)]
Urashima M, et al., 2019 [27]	417	Stages I to III	Vitamin D; *n* = 251	Placebo; *n* = 166	173/78	103/63	67 (61–75)	64 (58–71)	Digestive Tract Cancer (esophagus, stomach, small intestine, colon, and rectum)	2000 IU/day	- RFS - OS	- RFS: [77% vs. 69%; (hazard ratio, HR, for relapse or death, 0.76; 95% confidence interval, 95% CI, 0.50–1.14; *p* = 0.18)] - OS: [82% vs. 81%; (HR for death, 0.95; 95% CI, 0.57–1.57; *p* = 0.83)]
van Zweeden AA, et al., 2018 [28]	82	-	Chemotherapy + Folic acid and vitamin B12; *n* = 41	Chemotherapy; *n* = 41	33/8	33/8	61 (50–78) **	61 (35–82) **	Advanced esophagogastric cancer	Folic acid 450 µg/day; vitamin B12 1000 µg every 9 weeks	- RR - OS - TTP	- RR: [42.1% vs. 32.4%, (*p* = 0.4)] - OS: [median, 10.0 months vs. 7.7 months, *p* = 0.9)] - TTP: [5.9 months (1.4–33.5) vs. 5.4 months (1.4–30.9), (*p* = 0.9)]

* data are reported as mean ± standard deviation; ** data are reported as mean (range); Abbreviation is as follows: DCR, disease control rate; IL, interleukin; MMP, matrix metalloproteinase; ORR, overall response rate; OS, overall survival; PFS, progression free survival; RFS, relapse-free survival; RR, response rate; TTP, time to progression.

**Table 3:** Characteristics of other cancer patients in randomized clinical trials on nutraceutical supplements.

Author (Year)	Number of Patient	Stage Tumor	Nutraceutic Arm (Type; N)	Control Arm (Type; N)	Male/Female Nutraceutic (N)	Male/Female Control (N)	Age Nutraceutic Arm Mean ± SD	Age Control Arm Mean ± SD	Cancer Type	Nutraceutic Dosage	Outcome Included	Results
Datta M, et al., 2018 [29]	134	Stage I, II, III, or IV	Juice PLUS+; *n* = 72	Placebo; *n* = 62	61/11	52/10	58 (30–82) *	59 (41–82) *	Head and Neck Cancer	2 capsules in the morning and 2 in the afternoon/evening	- Measure of p27 expression and Ki-67 biomarkers associated with the development of second primary tumors	- Significantly higher serum levels of α-carotene (*p* = 0.009), β-carotene (*p* < 0.0001), and lutein (*p* = 0.003) but not of p27 (*p* = 0.23) or Ki-67 (*p* = 0.95) were observed
Demidov LV, et al., 2008 [30]	52	Stage III	FWGE + DTIC-based adjuvant chemotherapy; *n* = 26	DTIC; *n* = 26	15/11	15/11	50.4 ± 12.6	47.7 ± 13.9	Skin melanoma	8.5 g of FWGE granulate to dissolve in 150 mmL of water, orally once-daily	- PFS - OS	- PFS: 55.8 months vs. 29.9 months (*p* = 0.0137) - OS: 66.2 months vs. 44.7 months (*p* = 0.0298)
Mohseni H, et al., 2019 [31]	52	Stage I to III	Vitamin D; *n* = 26	Edible paraffin; *n* = 26	-	-	46.3 ± 9.5	47.7 ± 8.0	Breast cancer	Vitamin D3: 50,000 IU/week	- Measure of 25(OH) D3, TNF-α, TGF-β, and TAC based on VDR genotypes	- Vitamin D: (28 ± 2.6 to 39 ± 3.5; *p* = 0.004) - TAC: (48.9 ± 13.3 to 63.5 ± 13.3; *p* = 0.017) - Variations of TGF-β1 and TNF-α were not statistically significant
Shahvegharasl Z, et al., 2020 [32]	44	Stage I to III	Cholecalciferol; *n* = 22	Placebo; *n* = 22	-	-	44.1 ± 6.8	41.8 ± 7.5	Breast cancer	50,000 IU weekly of cholecalciferol	- Serum levels of VEGF-A, Ang-2, Hif-1, and hs-CRP	- Serum levels of Ang-2, Hif-1, hs-CRP, and Ang-2/VEGF-A were increased during the treatment period

* data are reported as median (range); Abbreviation is as follows: Ang, angiopoietin; DTIC, dacarbazine; FWGE, fermented wheat germ extract; Hif, hypoxia-inducible factor; hs-CRP, high-sensitivity C-reactive protein; OS, overall survival; PFS, progression free survival; TAC, total antioxidant capacity; VDR, vitamin D receptor; VEGF, vascular endothelial growth factor.

**Table 4:** Nutraceutical classes in cancer therapy.

**Nutraceuticals Classes**	Active ingredients of Nutraceuticals	Mechanism of Action	References
Polyphenolic Compounds	Flavones, Isoflavones, Flavonones, Flavonols, Phenolic Acids, Resveratrol, Curcumin	Alteration of signal pathways to remove cancer cells Block of the cell cycle Apoptosis	[33]
Carotenoids	Lycopene, α- and β-carotene, α-cryptoxanthin, Zeaxanthin, Fucoxanthin	Alteration of pathways leading to cell growth or cell death	[34]
Lipids and polyunsaturated fatty acids	Alpha-linolenic acid, Docosahexaenoic acid, Eicosapentaenoic acid	Regulation of metabolic pathways and inflammation Reduction of oxidative stress Alteration of the membrane composition and cell signalling pathways	[35]
Vitamins	Vitamin D, Vitamin B12	Promotion of cellular differentiation Inhibition of cancer cell growth Apoptosis Reduction of angiogenesis Decrease of tumor progression and metastasis	[36]

## Data Availability

The datasets generated and analysed in this study are available at the Embase, PubMed, and Web of Science websites.
